# A Course of Lectures on Dental Materia Medica and Therapeutics; Delivered at the College of Dentists in England

**Published:** 1863-12

**Authors:** Robert T. Hulme


### Selections.

*A Course of Lectures on Dental Materia Medica and Therapeutics; delivered at the College of Dentists in England.* By Robert T. Hulme, M. R. C. S., F. L. S. *Lecture III.*

Warm Fomentations.—The application of warm water to an inflamed part will often afford relief by relaxing the tissues, and thereby removing the tension and pain which accompanies the local disturbance. When inflammation has passed beyond that stage in which it may terminate by resolution, and must proceed to the formation of pus, warm fomentations are often used to hasten the process. Inflammation of the periosteum of the tooth frequently extends to the adjacent parts, and produces swelling of the cheek or other portion of the face. When this is the case, warm fomentations, either medicated or not, will be found to be serviceable and soothing to the patient. Care must, however, be taken that the fomentation of the part is not carried to such an extent as to induce the opening of an abscess on the exterior of the face instead of the interior of the mouth. Between the inflammatory and suppurative stage of alveolar abscess, rinsing the mouth frequently with warm water will hasten the process, and bring the matter more speedily to the surface. When there has been inflammation of the periosteum of a tooth previous to extraction, pain will frequently continue to be felt in the part for some hours after the operation; holding warm water in the mouth for a few minutes at a time will often afford relief. After the application of a leech to the gums, it is generally advisable to encourage the bleeding by the use of warm water; great caution, should, however, be observed in this matter, with regard to young children, in whom several instances have occurred of fatal hæmorrhage after the application of one or more leeches. If a leech attaches itself to the loose mucous tissue of the lip instead of the firm texture of the gum, it sometimes produces swelling and discoloration of the lip and cheek. These unpleasant effects are most apt to occur in females with a transparent delicate skin. When this happens, repeated fomentation of the part with warm water is the simplest and most efficacious remedy.

The temperature of the water should not be less than 62° F. and as much above that as is agreeable to the feelings of the patient. The efficacy of the fomentations is sometimes increased by the addition of a mild vegetable narcotic, such as the head and seeds of the poppy, this is especially the case when there is much pain and tenderness in the part.

Hot Air.—This mode of conveying heat to the general surface of the body is occasionally used in medicine; the only application of it in dentistry is for the purpose of drying the carious cavity of the tooth after the decay has been removed, preparatory to inserting the stopping material. For this purpose the following instrument has been contrived. It consists of a small blowpipe with a cylinder an inch long, and half an inch in diameter; this is placed down within two inches of the point of the instrument. This cylinder is either made of very heavy metal filled with wire, or something that will retain heat; on the other end is attached a stiff India rubber ball with an opening one and a half lines in diameter. By placing the thumb upon this opening, and making compression, a jet of air is forced through the point of the pipe, and the cylinder being previously heated, the temperature of the jet will correspond with that of the cylinder, and the velocity with which it is forced through the instrument. This jet thrown into a cavity that has been made as dry as possible by wiping down, makes a very perceptible change, the walls of the cavity becoming whiter than before. "This, we consider, the most desirable condition in respect to dryness that can be obtained."* I can not say I have found

* *A Practical Treatise on Operative Dentistry.* By J. Taft, p. 141, London, 1859.

this application as good in practice as it appears in theory. It is also more convenient to have the aperture in the ball placed opposite the end of the blowpipe, the latter can then be held between the fore and middle fingers, while the thumb compresses the ball. The termination of the blowpipe should not be too small, otherwise there is too great a resistance to the exit of the hot air.

If cotton wool, or tissue paper, is made use of for drying the cavity of the tooth, they answer the purpose better by having been previously heated, and thereby deprived of any moisture they may have received from the atmosphere of the room.

Actual Cautery.—The actual cautery is usually applied by means of an iron instrument furnished with a handle made of wood or of some non-conducting substance; the shape of the instrument varies according to the part to which it has to be applied and the purpose it is intended to fulfil. In general surgery, the actual cautery is sometimes employed for the suppression of hæmorrhage when it comes from a number of small vessels, or from a vessel to which a ligature can not be applied. The actual cautery is more frequently had recourse to in the removal of the upper or lower jaw, and in operations about the mouth, than in any other part of the body. Mr. Butcher, the surgeon of Mercer Hospital, Dublin, is a strong advocate for the use of the actual cautery in these operations. "In all instances," he says, "whether there be hæmorrhage or not, whether the growth be suspicious as to its character of malignancy or not, he invariably uses it, and that freely. If there be hæmorrhage, its application arrests the flow; if parts are suspiciously malignant it destroys contaminating radicle—tendency to recurrence; if no bleeding or malignance, its touch quickly arouses an inflammatory action, healthy in its action, vital in repair: by this rapid healthy change, destructive, diffuse erysipelatous inflammation are guarded against."

The removal of small tumors from the mouth is occasionally attended with very profuse hæmorrhage; in these cases the actual cautery will frequently be of service, and check the flow of blood should other measures fail. The following case serves to illustrate alike the application of the actual cautery and of cold:—

The patient was a female 43 years of age, of a sanguine, lymphatic temperament, and in good health. On the margin of the upper alveolus were two fleshy tumors resembling what is commonly termed epulis. The countenance was natural, with the exception of a slight elevation of the right half of the upper lip. On raising the lip two tumors were seen attached to the upper jaw. The smallest, about the size of a nut, was situated on the left side, and corresponded to the canine tooth and to the lateral incisor, behind which it formed its principal projection; its surface was irregular and granulated, of a purplish color, and elastic fungous consistence. There was no disposition to bleed, although the patient stated that on its first appearance the tumor bled freely with the least touch. The second tumor, three or four times as large, the size of half a walnut, was placed on the right side of the maxillary bone above the lateral incisor, the canine and the first bicuspis. Its base was spreading, and joined the gums inferiorly, which were softened and swollen, the tumor reached up to the canine fossa, to the middle line of the face and externally to the zygomatic fossa, it was of a wine-red color on its most prominent part. Its posterior surface rested on the maxillary bone, to which it was intimately attached. It was softer than the tumor on the opposite side. Neither of the tumors were painful. The neighboring teeth were loose.

M. Velpeau removed each tumor separately to avoid any unnecessary loss of the maxillary bone. The tumors were cut out with strong forceps. Immediately after the operation the blood poured from the wound on the right side, to such an extent that the patient, who had been operated upon in a chair, and without chloroform, fainted, and continued in that state for some minutes. This had the effect of arresting the hæmorrhage. As soon, however, as she came to herself, and was placed in bed, the bleeding returned in jets, and abundant. Pledgets of lint, saturated with perchloride of iron, held in place by the forcible closing of the jaws, and fragments of ice, were placed in the mouth, and frequently renewed. An hour and a half afterwards, the lint had become saturated with red blood, and made its way into the mouth. The lint was then removed, an artery tied, which appeared to be the alveolar, and as the blood appeared to come out in jets, the whole surface was cauterised by a red-hot iron—which arrested the hæmorrhage. The pledgets, saturated with the perchloride, were then placed in the wound.

The application of the gum lancet, or of a leech to the child's gums, is sometimes followed by a hæmorrhage which can only be arrested by the use of the actual cautery.

In operations upon the teeth themselves, the actual cautery has been proposed for two purposes; first, for destroying the pulp of the tooth, and secondly, for arresting the hæmorrhage which occasionally follows the operation of extraction.

The application of a red-hot wire to destroy the pulp of the aching tooth is a very old and popular remedy; but, at the same time, it is one of the worst means which could be suggested. We find, therefore, no writer on Dental Surgery of any authority recommending the use of it, thus Fox says: "Attempts have sometimes been made to destroy the nerve with the actual cautery, by introducing a red-hot wire; but I never have scarcely ever found this plan to be effectual; and as it always gives great pain, and sometimes produces an increase of inflammation, I think it is better never to recommend it. Indeed, all applications are very uncertain, and, therefore, if relief be not speedily obtained, it is advisable, to suffer pain once for all, by having the tooth extracted. That this remedy may, however, be occasionally successful when applied with resolution, we have the following proof afforded:—

"A friend of mine," says the writer, "tells me that, when at Cambridge, he was dreadfully tortured with pain in a carious tooth, and that one day, quite worn out with suffering, he broke off all the prongs but one of a dinner fork; the remaining one he heated red hot, and in that condition thrust it forcibly into the hole in the aching tooth. The pain, he says, ceased in a minute, and that he has not, from that time to this, a period of nearly thirty years, had a single twinge of toothache."

The effects of heat upon the body varies according to the temperature at which it is applied; thus, a white heat will immediately destroy the part, and without that amount of pain which is generally supposed, but a lower temperature, although it may ultimately cause its destruction, is accompanied by inflammation, a circumstance which would increase instead of alleviating the pain arising from the exposed and inflamed pulp of the tooth. It is evident that it is no easy matter to apply a wire at the requisite temperature to the interior of a tooth; for this reason the remedy can not be relied upon, and has been very properly rejected. This objection does not hold with regard to the actual cautery when applied by means of electricity, in the manner hereafter to be described.

In cases of hæmorrhage after the extraction of a tooth or from the surface of the gums, the propriety of using the actual cautery depends most materially upon the constitution of the patient. If the bleeding occurred in a person in whom the hæmorrhagic diathesis was present, little or no good is, I think, to be expected from the actual cautery. In a fatal case of hæmorrhage recorded by Mr. Blagden in the *Med. Chir. Trans.*, vol. viii., p. 224, the actual cautery was applied by Mr. Brodie, and succeeded in restraining the bleeding for the space of six hours; it then returned, and the cautery was twice repeated, but without success. In a similar case, published in the *Medical Gazette* for 1841, by Mr. W. A. Roberts, of Edinburgh, and in which the patient died on the twenty-third day, it is stated that on the day of the hæmorrhage, Dr. Hay was called in, and applied the actual cautery without benefit. Afterwards, with an iron better adapted, Mr. Roberts used it again, but with no better result. In applying the iron the patient started, and the under lip was slightly burned. From the wound thus produced, blood oozed freely for several days, a circumstance which conveys an important caution should the practitioner be induced to resort to the use of this remedy in any future case of a similar kind. In both these patients the hæmorrhagic diathesis was strongly marked, and it is only in such cases that we have cause ever to apprehend a fatal termination.

In all cases where the bleeding continues, the medical attendant should endeavor to ascertain whether the patient has ever before shown that he possessed the hæmorrhagic diathesis, and should that be the case, in conjunction with local he should combine constitutional treatment. We have seen in the above examples that the actual cautery was useless if not worse than useless. Let it also be added that in the first case, the carotid artery was tied for the purpose of cutting off the supply of blood, and the consequence was that the wound which was inflicted in the operation continued to ooze with blood, and thus became another fountain from whence the stream of life was passing away. Speaking of this subject, Dr. Richardson says: "I think, however, that too much exclusion has been often practised in these cases; and that in many of the fatal instances where death has occurred after three or four days, or even weeks, of hæmorrhage, that while too much care has certainly not been bestowed upon the local treatment, too little has been thought of in relation to general or systematic treatment. I would therefore suggest, I believe for the first time, that in cases of hæmorrhage of the kind I am describing, the internal administration of acids should be from the first considered. I will show the force of this recommendation. There is a form of hæmorrhage called hæmoptysis. In this case, as in the case of hæmorrhage from a tooth, the current of blood comes from a divided artery. Here, however, the divided vessel is in the lung, and out of reach; and if the physician, feeling the impossibility of local treatment, were to put his hand in his pocket, his patient would die much sooner than from any loss of blood from a dental artery; yet in vast numbers of cases, the patient is saved in hæmoptysis by the physician. How? By the simple administration of the mineral acids, or of acetate of lead. The effect here is through the system, but in its ultimate results it is localised, or local."

Hæmorrhage after tooth extraction occasionally takes place in persons who are otherwise apparently strong and healthy, and in whom the blood speedily coagulates. In these cases, should other means fail, recourse may be had to the actual cautery. If this is determined upon, care must be taken that the instrument is so shaped that it shall reach the seat of the hæmorrhage. For this purpose a conical-pointed piece of soft iron should be fixed in a handle made of some material which is a bad conductor of heat, such as ivory or wood. If the cauteriser is intended for one of the molar teeth, the end of it should be bent at a somewhat acute angle, taking care that the bent portion is sufficiently long to reach the extremity of the socket.

With regard to the upper bicuspids, the extracted tooth should be examined to ascertain whether the fang is bifurcated or not. If this should prove to be the case, it would be necessary to use a very narrow cauteriser, as the extremities of these bifurcated fangs are generally very pointed. In a case where the hæmorrhage came from any of the six front teeth, a straight conical-pointed instrument would be the best. Care should be taken that the instrument is applied to the part at the highest possible temperature which can be given to it. By this means the operation is rendered more effectual, and is attended with less pain.

Harris mentions two cases of hæmorrhage, in which he found it necessary to have recourse to the actual cautery; and as he makes no further comment upon them, one is led to conclude that the remedy was successful. The same writer records an instance in which an excessive and dangerous hæmorrhage following the extraction of teeth, occurred in three members of the same family—a lady and her two daughters—showing that this rare and serious complication of the operation may partake of an hereditary character. The cases in which hæmorrhage occurs, after the extraction of a tooth, may be divided into two series. In the one series, the patient possesses the hæmorrhagic diathesis; in the other, this diathesis is absent, and the patient may be either debilitated or plethoric.

The following is a summary of the principal cases I have been able to collect of severe hæmorrhage, or of death from bleeding after the extraction of a tooth:—

Mr. Blagden's case, already referred to, was a man, twenty-six years of age; the tooth, from which the bleeding came, was a second molar of the upper jaw. The actual cautery was applied, and the carotid artery tied. The hæmorrhagic diathesis was present, and the patient died on the sixth day.

Snell relates the case of an elderly person who had one loose tooth, a bicuspis, which was extracted; hæmorrhage ensued, and in spite of remedies he died on the day after the operation.

Mr. Robertson's case was a gentleman of middle age. The bleeding followed the extraction of a loose wisdom tooth in the lower jaw. He was rather of a full habit; the hæmorrhagic diathesis was present, and he died on the twenty-third day.

In a case related by Mr. Davenport, the man was twenty-three years of age, and the tooth a molar in the lower jaw. The patient was healthy; the bleeding continued for thirty-four hours and then stopped. A great quantity of blood was lost and the patient in considerable danger.

Harris' case was a female, aged fifteen years, and the tooth the second molar of the upper jaw.

One of the old French writers has recorded a case of death after extraction of one of the four front teeth. The patient was a female, and the gums attacked with scurvy.

Dr. Richardson met with an instance in which hæmorrhage occurred in a man after extraction of one of the lower molars, and continued for four days. The man was exhausted and the blood was coming away freely from the socket of the tooth. It was arrested by the application of dilute nitric acid, pledgets of lint, after thoroughly clearing out the socket, by syringing it with warm water.

Mr. Mason Good has published the following instructive case, showing the danger into which a patient may be brought from the improper application of the means usually employed for arresting hæmorrhage after tooth extraction:—"I was, not long ago, requested to see a young man who had been profusely bleeding from the gum and socket of an extracted tooth five days without cessation and without sleep, till his wan cheeks and faint emaciated frame seemed to indicate that he had scarcely any blood left in his vessels. He was so weak as to be incapable of rising from his bed or taking food, and his stools, from the quantity of blood he was perpetually swallowing, had all the appearance of a malœna. On opening his mouth, I found it crammed full of lint and wadding, one piece having every hour been added to another without a removal of the preceding, lest the hæmorrhage should be increased; whilst the blood in which the wadding was soaked, and which had remained in the socket and over the gums for so long a period, was become grumous, putrid, and intolerably offensive.

"I first removed the whole of this nauseating load from the patient's mouth, and gave him some warm brandy-and-water to wash it with. I next directed him to take a goblet of negus, with a little biscuit sopped in it, a part of which he soon contrived to swallow. The bleeding still continued; but, as I had little doubt that this proceeded entirely from a want of power in the lacerated arteries to contract, I applied no pressure of any kind, but applied a gargle of equal parts of tincture of catechu and warm water, and then hæmorrhage soon ceased."

From the consideration of these cases, we learn the following important facts: First, wherever death has ensued, and we possess a full knowledge of the circumstances, the patient has had the hæmorrhagic diathesis, or was the subject of scurvy: in the latter case the bleeding did not come so much from the socket of the tooth as from the surface of the gums. Secondly, the bleeding lasts for several days, and ample time is allowed for the administration and operation of internal remedies.

In cases where the hæmorrhagic diathesis is present, we ought, therefore, to rely rather upon constitutional than local remedies, but when the patient is plethoric, or only of a weak constitution, local remedies may generally be depended upon to arrest the bleeding. Should the various styptics, to be hereafter mentioned, in conjunction with pressure, fail to arrest the bleeding, then recourse might be had to the actual cautery. If the patient is anæmic or debilitated, diluents must be avoided, tonics and a nourishing diet should be ordered, and even the addition of a small amount of stimulants may be advisable.

Some years ago, M. Cloquet proposed to make use of the actual cautery for the purpose of curing congenital fissure of the soft palate. This "plan consists of cauterising the angle of the fissure for a very limited extent, and allowing it to cicatrise; then repeating the operation again and again, till the contraction following each cicatrization has closed the whole fissure. The cautery should be a small iron at a black heat. M. Cloquet has tried this method upon four patients at the date (1855) of the publication of this paper, with success." At the meeting of the Academy of Sciences of Paris, on the 21st of May, 1860, M. Cloquet brought forward a case, treated by Professor Benoit, of Montpelier. The child was eleven years old, the soft palate was completely cleft, and all the usual symptoms were present; the treatment lasted nineteen months, with two rather long interruptions. The whole cleft was now united, save that of the uvula, and the result was obtained by thirty-three cauterizations, fourteen with the acid nitrate of mercury, and nineteen with the solid nitrate of silver. A slight nasal pronunciation still exists, being the result of habit. M. Benoit intended to apply the same treatment to the uvula.

This plan of treating fissures of the soft palate, either by the actual or chemical cauterization, although tedious in its application, would appear to be deserving of greater attention than it has hitherto received from surgeons in this country.—*Dental Review.*

Deaths from Chloroform.—The London *Lancet* of Nov. 7th contains full reports of two new cases of deaths from chloroform inhalation in private practice, and the week previously noticed two which had just occurred in London hospitals. We take the following extracts:—

"At the inquest, Dr. Blackmore said—I saw the deceased for the first time on Sunday night; she was suffering from fistula. On Monday I called, and told Mrs. Luther that although I did not think it necessary to bring a second surgeon, I would do so if she wished it. She left the decision to myself, and I decided on not bringing any one with me. The patient was lying in bed, and appeared pretty well I ordered castor oil and a little brandy-and-water. Before I commenced the operation I gave her about a tablespoonful of raw brandy. I then poured a small quantity of chloroform on a handkerchief, which I placed on her face; she inhaled it freely and was soon under its influence, but struggled slightly while she was insensible. The whole quantity of chloroform taken was four drachms and a half. She was insensible for about six or seven minutes. The chloroform was quite pure, and of the usual specific gravity. As she became insensible I commenced the operation, which lasted two or three minutes. The handkerchief was removed before the operation. At the termination of the operation I found she had not revived; her respiration became gradually slower, and ceased in about three minutes. I applied cold water to the face, administered sal volatile, used the galvanic apparatus, and had recourse to Dr. Marshall Hall's plan for procuring artificial respiration, without effect. I am constantly in the habit of using chloroform, and occasionally without an assistant, according to the nature of the operation. Unless there are peculiar reasons, it is not necessary to have the assistance of a second surgeon. From her previous history there were no symptoms that led me to infer that there was the slightest danger. Her heart did not appear to be diseased. She suffered occasionally from indigestion. * * * * * *

"The second case occurred in the practice of Mr. John Gay, surgeon, of Finsbury place, Finsbury, also in the person of a young woman, aged 16.

"At the inquest, Mr. Gay said—I was consulted about six weeks ago for a hare-lip and cleft palate and enlargement of the tonsils. I removed the tonsils six weeks ago, not under chloroform. Then the palate was to be repaired. After the operation on the tonsils the deceased became generally well. On Wednesday I went to her house to operate on the palate. Mr. Worley helped me. It was not under chloroform. After commencing the operation her courage failed her, and she would not allow me to proceed. At that portion of the treatment it was agreed that the operation should be deferred. On Thursday she came to my house to have her lip operated on, and Mr. Worley came to assist me. Mr. Worley is a surgeon of Hoxton, and a very skillful man. He has administered chloroform under my superintendence. The deceased appeared in good health and spirits, and sat down in a chair. Mr. Worley assisted me in the operation on a child, and it was all right. We used an inhaler for the deceased, but it had not the desired effect, and a piece of lint saturated with chloroform, was applied to her nostrils. In three or four minutes the usual spasms which precede the loss of sensibility were apparent, and I was waiting for complete insensibility, when I discovered that the pupil dilated rapidly; she fell back, and her face became pale and her lips bluish. I put my hand to her pulse, and it was gone then—there was no life. I immediately tried every means with artificial respiration for half an hour, without success. The mother, my servant, Mr. Worley, and myself were in the room. Mr. Worley did not attend to her pulse continuously. It was not necessary in my judgment. While he was administering the chloroform, I was standing close by watching the effect of the chloroform, and making any suggestion that might arise. I had felt the pulse more than once. I do not think it necessary for the same person to notice the pulse who administers the chloroform. It is constantly the practice to administer chloroform without noticing the pulse. I was not holding her pulse at the time when she went off. I think an occasional feeling of the pulse quite sufficient. I had listened to her heart before. I know it was not diseased. This is not so good a criterion as other means—such as watching the pupil of the eye and feeling the temporal artery. * * * *

"The learned coroner, in summing up the evidence, dwelt in strong terms upon the omission of medical men continuously to watch the pulse; for, he said, had the pulse been continuously attended to, it was probable that the life of the deceased might have been saved. From his own personal experience he could say that it was usual for the administrator to hold the pulse. He could not recommend the jury to return a verdict of manslaughter, for it was evident that it was an error of judgment.

"After a long consultation, the jury returned a verdict 'That Ellen Smith had died from the effects of chloroform administered previous to an operation, and that there was no blame attached to the medical men.'"

In its editorial comments on these cases the Lancet says: "We consider that in *all* cases the chloroform ought to be administered by a second person, whose sole business it is to attend to that duty. We deem it to be a most important function of that person to carefully watch the face of the patient, note his respiration, and above all attend carefully to the state of the pulse. The results of investigations certainly tend more and more to confirm the opinion that these deaths by chloroform are heart-deaths, and the observation of the pulse is therefore a matter of the last importance. Finally, we believe that chloroform ought to be administered as far as possible in a definite relation of percentage to the atmospheric air supplied at the same time; and no apparatus yet devisod seems to fulfill the indication so well as that of Mr. Clover, which is in use at the University College Hospital."—*Boston Med. & Surg. Journal.*

